# Immunotherapy: A Case Series

**DOI:** 10.7759/cureus.19726

**Published:** 2021-11-18

**Authors:** Tuong Vi C Do, Mythili Kanthi Gudipati, Subramanya Shyam Ganti, Jayaramakrishna Depa, Kamlesh Sajnani

**Affiliations:** 1 Internal Medicine, West Anaheim Medical Center, Anaheim, USA; 2 Internal Medicine, Harlan ARH Hospital, Harlan, USA; 3 Internal Medicine: Pulmonology Critical Care, Harlan ARH Hospital, Harlan, USA; 4 Internal Medicine: Nephrology, Harlan ARH Hospital, Harlan, USA; 5 Internal Medicine: Oncology, Harlan ARH Hospital, Harlan, USA

**Keywords:** hypophysitis, nephritis, thyroiditis, transminitis, pneumonitis, nivolumab, pembrolizumab, immunotherapy

## Abstract

Immunotherapy is on the rise as a treatment option for advanced melanoma, non-small cell lung carcinoma, renal cell carcinoma, and melanoma among others. It consists of two main classes being cytotoxic T lymphocyte antigen 4 (CTLA 4) inhibitors and programmed cell death 1 (PD 1) inhibitors. We report a case series of four patients who were started on either pembrolizumab or nivolumab for the treatment of melanoma or lung cancer. While on immunotherapy, they developed various side effects related to the immunotherapy including pneumonitis, transaminitis, thyroiditis, nephritis, and hypophysitis. To treat this complication, immunotherapy must be discontinued or held with immunosuppressant initiation as treatment. Most often the immunosuppressant of choice is steroids. After symptoms improve, patients can decide along with the clinician on restarting or completely stopping immunotherapy. Within our case series, three of four patients had resolutions of their symptoms with steroid treatment with one who was lost to follow up. Of the three patients who were being followed up, one had a relapse of side effects after resuming immunotherapy and decided against further treatment with immunotherapy. Another patient is doing well resuming immunotherapy on a daily dose of steroids. The last patient decided to not continue with immunotherapy after experiencing a flare of his symptoms when he was being treated since he missed a few doses of steroids. Further research is needed about the risk of flares of complications when resuming immunotherapy alone or with immunotherapy and steroid treatment.

## Introduction

Immunotherapy builds up the immune system to fight various diseases and has become a promising and revolutionizing treatment in certain cancers [1]. Immunotherapy is on the rise as a treatment option for various advanced malignancies, including melanoma, non-small cell carcinoma, and renal cell carcinoma [2]. They work by blocking the function of immune checkpoints allowing for antitumoral responses. Immunotherapy can be further classified as cytotoxic T lymphocyte antigen 4 (CTLA 4) inhibitors and programmed cell death 1 (PDL 1) inhibitors. The CTLA 4 inhibitor class consists of ipilimumab and tremelimumab. Within the PDL 1 inhibitor class, it consists of nivolumab, pembrolizumab, cemiplimab, atezolizumab, avelumab, and durvalumab [2]. PDL 1 inhibitors cause dose-independent adverse effects [3]. Immune checkpoint inhibitors have a wide spectrum of side effects described as immune-related adverse events (irAEs) and consist of colitis, vitiligo, thyroiditis, hepatitis, pneumonitis, transaminitis, nephritis, and hypophysitis [2-4]. These irAEs lead to temporary or permanent discontinuation of immunotherapy based on type and grade of side effects. Herein, we present four patients who had received either pembrolizumab or nivolumab and developed irAEs.

## Case presentation

Case 1

**Figure 1:**
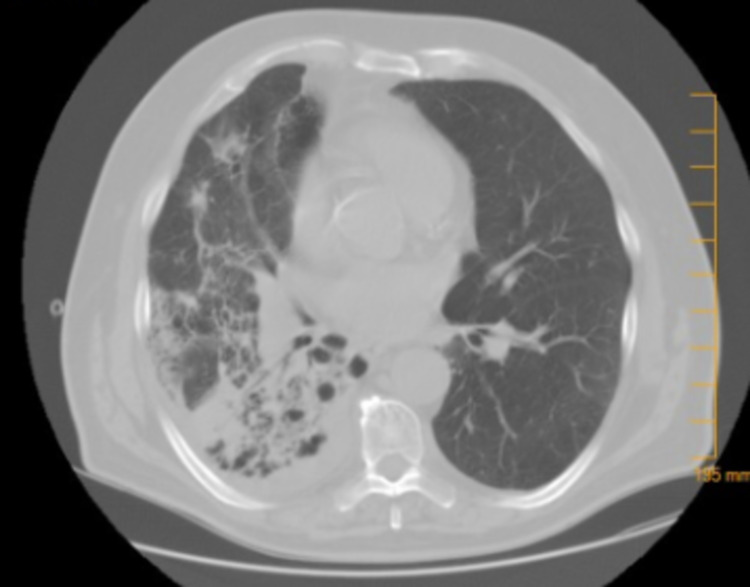
Upon the first diagnosis of pembrolizumab-induced pneumonitis based on interval interstitial infiltrate in the posterior segment of the right upper, middle, and lower lobes

**Figure 2:**
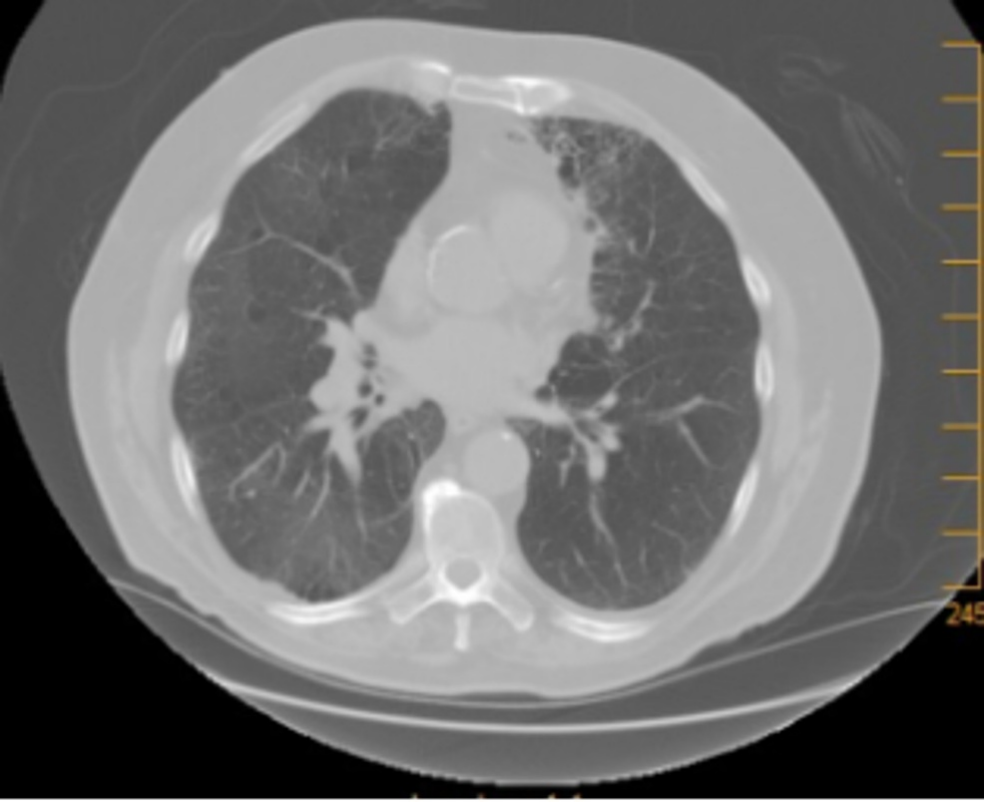
Three months later on follow-up after discontinuation of steroid taper

A 77-year-old male, with a history of chronic obstructive pulmonary disease (COPD), diagnosed with left shoulder melanoma in 2015, treated by wide excision along with radiation therapy, had a relapse locally at the original site with lung metastasis (stage IIA; pT4, N0, M1b) and was BRAF negative in 2017. He was started on pembrolizumab in August 2017 with a cycle of every three weeks for treatment. Two years later, in 2019, he presented with generalized weakness, productive cough that was clear, acute on chronic worsening dyspnea, and diarrhea for the past week, with his last dose of pembrolizumab three weeks back. He denied any fever, orthopnea, or lower extremity swelling. He admitted to paroxysmal nocturnal dyspnea. He uses 3 L of home oxygen presently requiring high-flow oxygen. On exam, he had audible crackles on the right side along with diminished lung sounds throughout the right lung. His chest X-ray (CXR) showed right middle and lower lobe infiltrates along with interstitial changes. The white blood cell count was normal. His chest computerized tomography (CT), as seen in Figure 1, showed severe bullous changes in bilateral lungs, a small right pleural effusion, a spiculated nodule in the left lower lobe measuring 1.1 cm by 2.2 cm, thickening of the interlobular area, and interval interstitial infiltrate in the posterior segment of the right upper lobe, right middle lobe, and right lower lobe. At this time, his pembrolizumab was discontinued, and he was started on broad-spectrum antibiotics including atypical coverage. Methylprednisolone was also started. He underwent bronchoscopy with bronchoalveolar lavage to rule out infection, which showed mucosa inflammation of the right lower lobe, right middle lobe, and the anterior segment of the right upper lobe with dark yellow mucus plug seen. Bronchoalveolar lavage (BAL) cultures, viral panel, and Pneumocystis carinii smear were negative. He was discharged home in October 2019 on prednisone 40 mg b.i.d. with a plan to taper down over the next six weeks in the outpatient setting. He developed two consecutive episodes of secondary spontaneous pneumothorax in which he required chest tube placement and missed at least one week of his steroids, which lead to a flare-up of his pneumonitis and getting readmitted to the hospital in November 2019. He was restarted on steroids and followed up with an outpatient pulmonologist where his steroids were weaned in the next eight weeks. Repeat chest CT, as seen in Figure 2, done three months later showed near resolution of the interstitial infiltrate in the right upper, middle, and lower lobes and unchanged left lower lobe spiculated nodule. In June 2021, he was tapered down to prednisone 2.5 mg daily. His respiratory status was stable, with chest CT showing resolution of pneumonitis. In September 2021, he expired in his sleep.

Case 2

**Figure 3:**
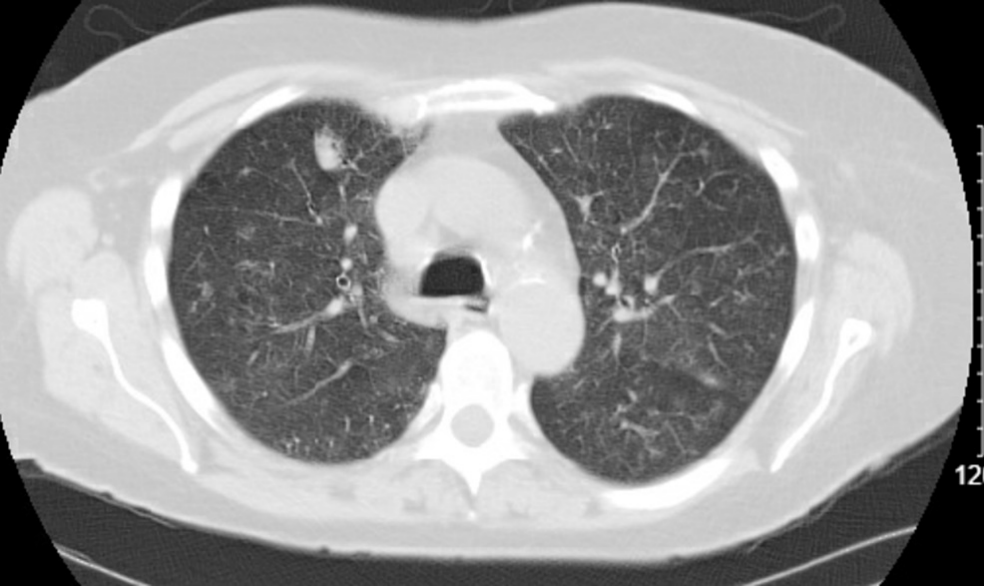
Pulmonary nodule and diffuse ground-glass changes in February 2020

**Figure 4:**
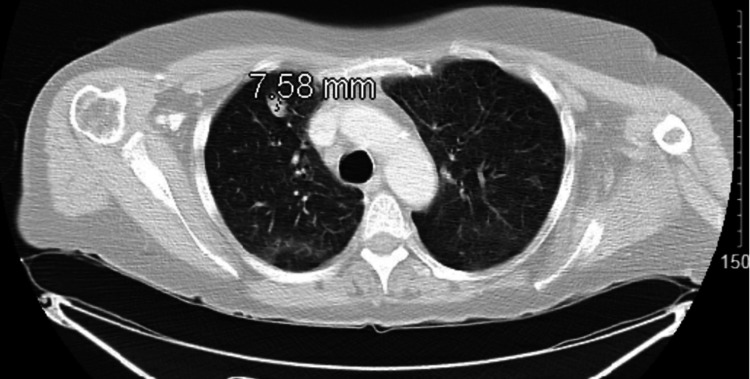
Pulmonary nodule in January 2020

A 64-year-old female undergoing chemotherapy and radiation for malignant mucosal lentiginous melanoma of the left gingiva (stage III; pT3, N1, M0) presented in February 2020 with fatigue and worsening shortness of breath for the last two months. She had a left modified radical neck dissection with resection of the left oral cavity. She then underwent 30 treatments of external beam radiation. After radiation was completed, she was started on nivolumab 240 mg IV every two weeks in August 2019. In January 2020, her nivolumab was held due to worsening transaminitis and thyroiditis, which presented as hypothyroidism and then hyperthyroidism after starting levothyroxine. CT abdomen was done at that time, which showed increased heterogeneous attenuation of the liver. She was started on prednisone 40 mg daily. With her history of being a current smoker of 1 ppd for the last 30 years, she admitted to a productive cough that was clear in nature, chest tightness, and orthopnea but denied paroxysmal dyspnea and fever. Chest CT without contrast, done in February 2020, as seen in Figure 3, showed multiple pulmonary nodules with some interstitial/ground-glass changes in the upper lobes bilaterally, right middle lobe, and right lower lobe, which were worse than her previous chest CT two weeks prior, as seen in Figure 4. She was diagnosed with nivolumab-induced pneumonitis. Her lab work showed a very low thyroid-stimulating hormone (TSH) level of < 0.07 along with her free triiodothyronine (T3) being normal, free thyroxine (T4) of 4.6, and negative thyroid peroxidase antibody (TPO) antibody. Levothyroxine was then stopped. She still had transaminitis. She was started on ceftriaxone and azithromycin for possible pneumonia. A higher intravenous dose of steroids was initiated. She underwent bronchoscopy with BAL with findings of erythematous bronchial mucosa of the right lower lobe. BAL results were negative, including a viral panel, Gram stain and culture, and fungal stain and culture. After increasing the steroids, she felt better, and her breathing improved. She was discharged on prednisone 60 mg daily and was recommended to follow up on an outpatient basis with oncology and pulmonology. Unfortunately, she did not follow up with either oncology or pulmonology and was lost to follow-up.

Case 3

**Figure 5:**
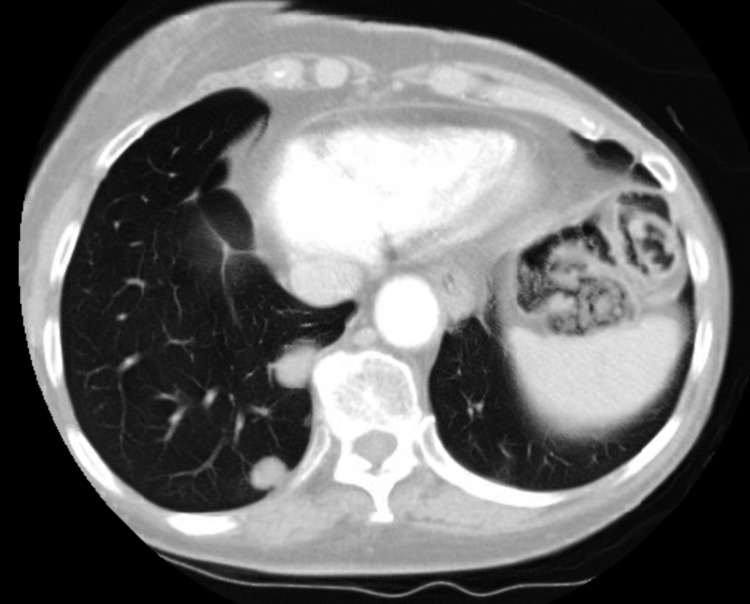
Multiple nodular masses in the right lower lobe in January 2019

**Figure 6:**
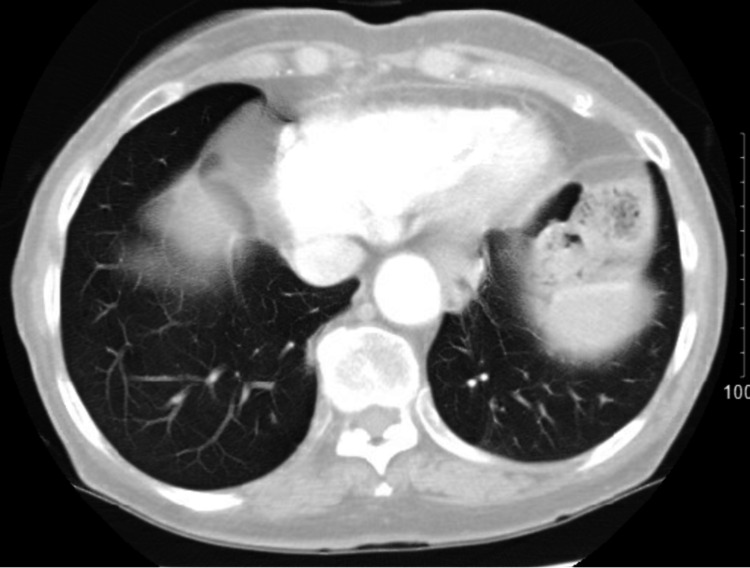
Resolution of right lower pulmonary masses in October 2019

**Figure 7:**
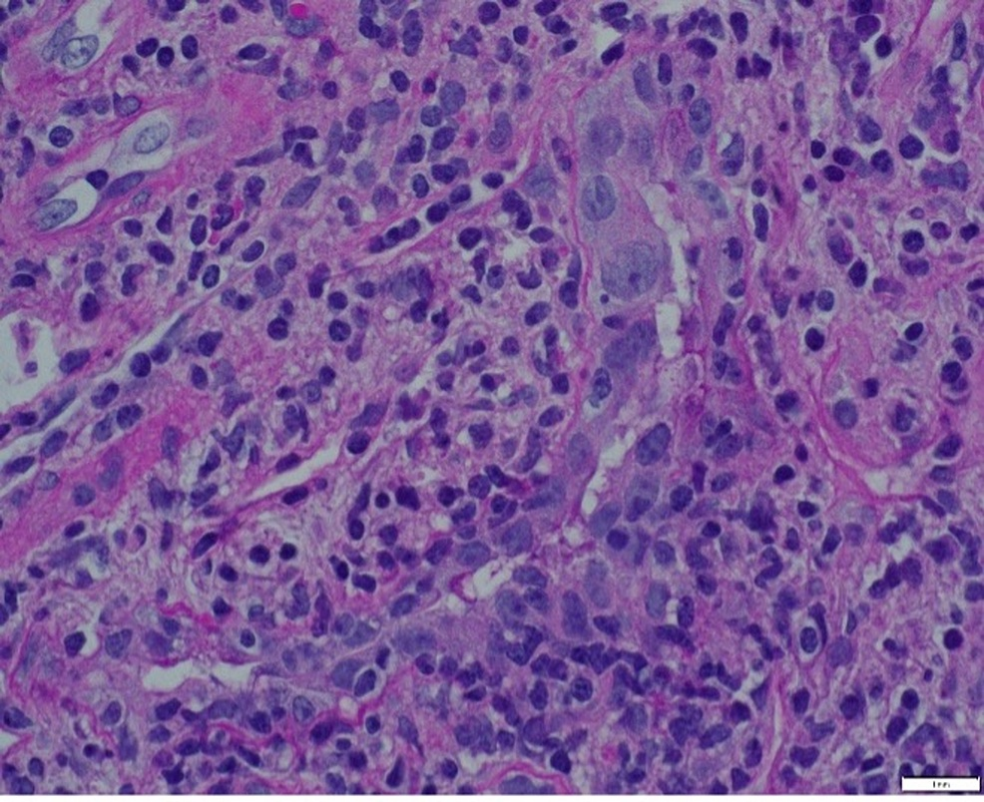
Moderate diffuse interstitial inflammation of mainly lymphocytes and monocytes with moderate multifocal lymphocytic tubulitis

A 65-year-old female diagnosed with non-small cell adenocarcinoma of the right middle lobe, as seen on chest CT (stage IIA, pT2B, pN0, M0) in 2017, underwent right middle lobectomy and mediastinal node dissection three months later. The pathology report showed moderately differentiated adenocarcinoma with visceral pleural invasion and transcription termination factor 1 positive. She completed carboplatin/Alimta in 2018. In January 2019, her repeat chest CT (Figure 5) showed multiple nodular masses in the right lower lobe where her original lung cancer was. A positron emission tomography (PET)/CT scan was done, which showed subpleural nodules with moderate fluorodeoxyglucose (FDG) activity, indicating relapse. At that time, she had declined a biopsy. She was started on palliative chemotherapy of carboplatin, taxol, and pembrolizumab in March 2019 and finished the regimen in May 2019. A repeat chest CT done in May 2019 showed a decreased size of her right lower lobe pulmonary nodules without evidence of progression or new distant lesions. She started pembrolizumab for maintenance therapy in June 2019. Then, her chest CT in October 2019 (Figure 6) showed complete resolution of the pulmonary nodules with no evidence of progression or new lesions. However, in March 2020, her creatinine (Cr) level started to increase to 3.07 even though she was asymptomatic with no lower extremities edema. Her baseline Cr level was around 1.0-1.2. Her Cr level had increased to 5.53 in April 2020 and further workup was initiated. Her protein: Cr ratio was 1315, no monoclonal protein was identified, and antinuclear antibodies (ANA) and vasculitis profile were negative. Complement levels were normal. She subsequently underwent a kidney biopsy with pathology (Figure 7) showing acute and chronic interstitial nephritis grade 3 and tubulitis. Further workup with the kidney was unremarkable. Her pembrolizumab was held toward the end of March, and she was started on a prednisone 40 mg taper. Her Cr did improve to 1.19, close to her baseline, in May 2020. She completed the steroid taper in July 2020. With the improvement of her Cr, she resumed pembrolizumab in July 2020; however, her Cr level started to increase up to 2.09. She was restarted on prednisone 20 mg daily, and after discussion with oncology, a decision was made to stop pembrolizumab indefinitely. In September 2020, she was tapered down to 10 mg daily. She eventually expired a few months later.

Case 4

A 53-year-old female was diagnosed with metastatic melanoma (cTx, pN1b, M1) in February 2019. She originally presented with left inguinal swelling of four months duration in October 2018. Her ultrasound at that time had only shown a prominent left inguinal lymph node for which core biopsy was done in February 2019, confirming it to be melanoma. Her abdomen pelvis CT also showed bilateral inguinal lymph nodes with a lesion at the splenic lower pole. Follow-up chest CT had shown a neoplastic solid mass in the spleen. She had a left inguinal node dissection in March 2019, which was positive for melanoma with 1/22 nodes positive, the largest one being 5 cm. The PET/CT scan seen in Figure 8 in March 2019 was positive for multiple discrete hypermetabolic foci within the spleen without abnormal foci of increased fluorodeoxyglucose (FDG) in either the liver or lungs. She underwent splenectomy in April 2019 with pathology reporting multiple foci of metastatic melanoma. She was started on pembrolizumab in April 2019. However, in January 2020, she presented to outpatient oncology with progressive generalized weakness, extreme fatigue, lethargy, myalgia, poor appetite, weight loss, and mood changes over a period of 6-8 weeks. Labs in February 2020 showed low free cortisol of 0.2 ug/dL and low adrenocorticotropic hormone (ACTH) of < 1.1 pg/mL. Her luteinizing hormone, follicle-stimulating hormone, and prolactin were normal. She was diagnosed with pembrolizumab-induced hypophysitis, and the decision was to hold her pembrolizumab. She was started on prednisone 1 mg/kg/day with gradual tapering to the maintenance dose of 10 mg daily. Repeat cortisol level done in March 2020 was within the normal range of 3.0 ug/dL. Her cortisol response to adrenocorticotropic hormone (ACTH) was also normal at 3.2 ug/dL. She then resumed pembrolizumab in March 2020. Her repeat chest CT in May 2020 was negative for any recurrent cancer. She is currently tolerating pembrolizumab, reporting good energy levels without any fatigue, myalgia, or mood changes while still on prednisone 7.5 mg daily. She finished her treatment course of pembrolizumab in April 2021. With her latest follow-up in August 2021, she did not have any recurrent disease and is in remission, remaining on prednisone 7.5 mg daily. 

**Figure 8:**
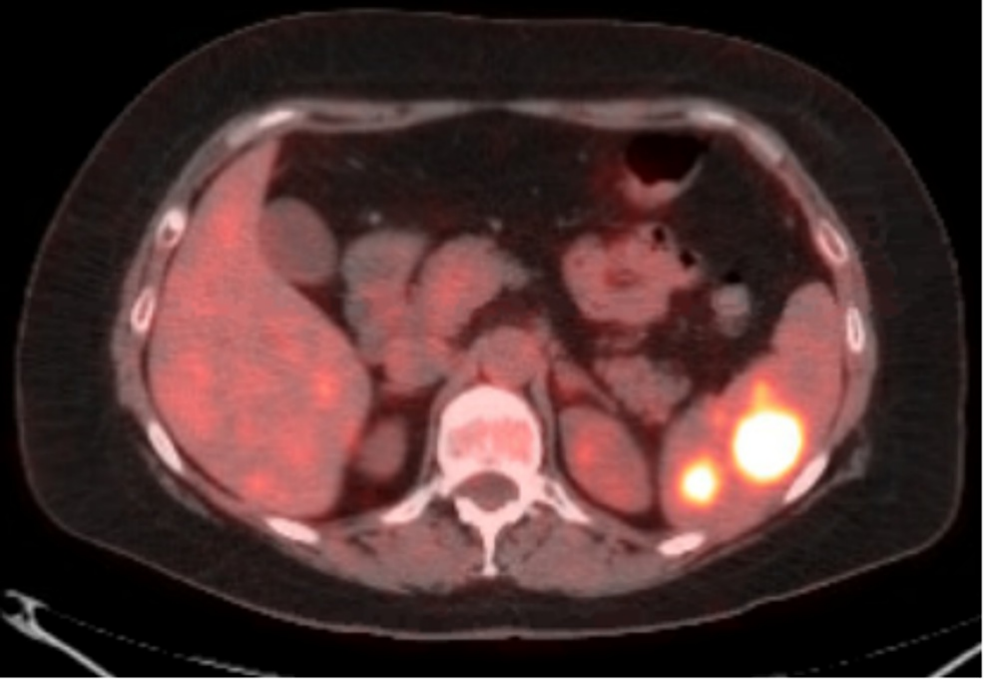
PET scan showing increased FDG uptake in the spleen PET: positron emission tomography; FDG: fluorodeoxyglucose

## Discussion

Immunotherapy approved for various malignancies can cause various adverse effects depending on the agent itself. Our case presentations were focused on the PDL-1 immunotherapies. They function by blocking the programmed death-ligand 1 pathway, allowing cancer cells to hide from the immune system. Nivolumab has been approved for advanced melanoma, non-small cell lung carcinoma, renal cell carcinoma, head and neck cancer, Hodgkin lymphoma, gastric cancer, colorectal, esophageal, hepatocellular carcinoma, mesothelioma, and urothelial carcinoma [2,5]. Pembrolizumab has been approved for melanoma, non-small cell lung carcinoma, cutaneous squamous cell carcinoma, endometrial, hepatocellular carcinoma, Hodgkin's, Merkel cell carcinoma, renal cell carcinoma, urothelial carcinoma, and non-Hodgkin lymphoma, among other carcinomas. Although immunotherapy is a treatment option, they cause various adverse effects that often interrupt their current treatment. The side effects of immunotherapy can range from the head down, including the pituitary, thyroid, lungs, liver, and kidneys.

Hypophysitis is inflammation of the pituitary gland and is more commonly seen in those who are on CTLA antagonists rather than PDL 1 inhibitors [3,6]. However, our patient in Case 4 was on a PDL 1 inhibitor and developed hypophysitis. The mechanism of action of this adverse effect is due to the ACTH hormone. With the loss of ACTH, secondary adrenal insufficiency ensues for decreased cortisol action [6]. Symptoms are often headache, fatigue, and hypocortisolism. Our patient had, in addition to fatigue, progressive generalized weakness and mood changes. MRI imaging can be done to diagnosis hypophysitis. MRI of the brain findings of hypophysitis included moderate enlargement of the pituitary, enlargement of the stalk or infundibulum, and homogenous contrast enhancement. After steroid treatment, MRI findings would show a decrease in pituitary volume [3]. However, our patient had a brain MRI done a year prior to starting pembrolizumab, and the oncologist did not feel that a brain MRI needed to be done, just held her treatment, and started her on the steroid taper. Her symptoms had resolved with her cortisol level normalizing so her pembrolizumab was resumed while on maintenance steroid with no further symptoms of hypophysitis.

Thyroiditis is inflammation of the thyroid that can occur within the first three months on average [5]. Destructive thyroiditis is correlated with positive anti-thyroglobulin antibodies or TPO antibodies prior to treatment of immunotherapy [5]. It is associated with suppressed TSH levels with an elevated free T3 and/or free T4 and no TSH receptor antibodies [2,5]. The presentation can vary with some patients having thyrotoxicosis, with eventual normalization of the thyroid function. Thyrotoxicosis, however, can progress to hypothyroidism for which levothyroxine has to be started [5]. It is suggested that the severity of thyroiditis correlated with titers of pre-existing anti-thyroid antibodies [5]. Since our patient in Case 3 did not have TPO antibodies with normal free T4, her levothyroxine was discontinued. During acute thyroiditis, treatment is stopping the offending agent. After the thyroid function recovers, reintroduction of the offending agent did not further exacerbate thyroid dysfunction as seen in previous case reports [5]. Unfortunately, the patient did not follow up, so her current thyroid levels are unknown currently.

Pneumonitis is inflammation of the lungs and was described in Cases 1 and 2. It has a widely variable onset ranging from nine days to 19 months after initiation of therapy [7]. It can present as a persistent cough, dyspnea, tachypnea, and even hypoxia. CXR can often show interstitial infiltrates. The best diagnostic imaging modality is high-resolution CT, which confirms the diagnosis [8]. Based on CT, pneumonitis is classified as most to least common as cryptogenic organizing pneumonia (COP), nonspecific interstitial pneumonia (NSIP), hypersensitivity pneumonitis, and acute interstitial pneumonia (AIP)/acute respiratory distress syndrome (ARRDS) [3-4,9]. The grading system also classifies from the highest grade to the lowest as AIP/ARDS, COP, and then NSIP and HP [9]. A perilymphatic nodular pattern has been seen in one case as a bilateral, diffuse, well-defined, small, glandular shadow along with thickened interlobular septa [4,10]. Most often, it affects the lower lungs rather than the middle lungs and the upper lungs are the least likely [3,9]. Bronchoscopy and BAL can be done if the diagnosis is not confirmed by CT scan and to rule out infections [8]. Pathology often shows acute inflammation, reactive pneumocytes, and negative cultures [4]. The two pneumonitis patients we presented did have bronchoscopy with BAL, in which negative cultures were obtained. For low-grade pneumonitis (Grade 1), close observation is the best treatment option. For high-grade pneumonitis, treatment is to hold the offending agent along with the initiation of steroids. Both of our patients had high-grade pneumonitis, and their immunotherapy was held and they were started on steroids. The typical time for complete resolution with steroid taper was, on average, three to six weeks [8]. The readministration of immunotherapy after the resolution of pneumonitis can start a pneumonitis flare [9], which is a common occurrence with the same prior symptoms along with imaging findings. The readministration of a steroid taper will resolve the pneumonitis again. As seen in our patient in Case 1, he did develop a pneumonitis flare when he missed a week of steroids. However, for any pneumonitis that is not resolved with steroids, other immunosuppressant therapy like infliximab, mycophenolate mofetil, or cyclophosphamide can be added [10]. With our patient in Case 2 who was lost to follow up, it is unknown if her pneumonitis resolved with the steroid taper prescribed on an inpatient basis. However, for our patient in Case 1, pneumonitis completely resolved based on CT imaging after finishing a more extensive steroid taper since it was complicated with a pneumonitis flare.

Adverse effects of the gastrointestinal (GI) system, colitis, hepatitis, and pancreatitis, have also been reported. Hepatitis is seen as elevated transaminases with or without bilirubinemia [3]. Most are asymptomatic while others can have fever, fatigue, changes of stool color, and jaundice [3]. With our patient, she did have fatigue and was found to have transaminitis. Imaging is often done by CT or ultrasound. Based on previous case reports, CT is preferred, which often shows hepatomegaly, periportal edema, periportal lymph nodes, and attenuated liver parenchyma [3]. Treatment is with steroids while holding the offending agent. After steroids, hepatomegaly and periportal lymphadenopathy are usually resolved on imaging [3]. However, our patient in Case 2 failed to follow up with oncology, and no further lab work or imaging has been done.

Renal injury caused by immunotherapy often presents as acute tubulointerstitial nephritis (AIN). PDL1 immunotherapy often causes kidney injury later than CTLA 4 immunotherapy reported as three to 12 months and two to three months, respectively [6]. It is associated with hyperkalemia, an increase in Cr, and pyuria [6]. Hematuria, eosinophilia, and worsening hypertension can also be seen [11]. A kidney biopsy is often needed, which confirms AIN or podocytopathy [6]. AIN is seen as lymphocytes with varying degrees of plasma cells and eosinophils [11]. The grading of AIN is based on Cr levels. With our patient in Case 3, her serum Cr was 1.5-3x; her baseline classifying her AIN as Grade 3, so her immunotherapy was held with the initiation of steroids. Treatment is discontinuing the offending agent with steroids for an average of one to three months. Steroid treatment is often started in those with Grade 3 or higher with serum creatinine > 3x the baseline [12]. For those who do not respond to steroids, dialysis is required in addition to steroids, which can lead to partial remission. Full recovery is when serum Cr is < 0.35 mg/dL above the baseline value while partial recovery is serum Cr > 0.35 mg/dL but less than twice the baseline value [11]. Our patient did have full recovery upon discontinuation of pembrolizumab. However, she did resume pembrolizumab and developed worsening Cr levels. Therefore, she was started on prednisone taper again, and she elected to stop treatment with pembrolizumab.

## Conclusions

In conclusion, immunotherapy is preferred over chemotherapy and/or radiation for various malignancies since it builds up the immune system. However, they do lead to adverse effects that can affect the pituitary glands, lungs, thyroid, liver, and kidneys among others. This case series signifies that clinicians with patients on immunotherapy need to understand the common complications of immunotherapy to stop it and start immunosuppressants for the patient’s symptoms to improve. The most common immunosuppressant used is steroids. After resolution occurs, the decision to restart the immunotherapy can be made jointly with the patient and clinician. The risks versus benefits play a factor in this decision. As seen in previous cases, resuming immunotherapy can or cannot lead to further flares of adverse events. More studies are needed to determine the risk of further flares when restarting immunotherapy while on a maintenance dose of steroids.
